# Assessment of prescription writing skills among dental house officers: A multi-center study

**DOI:** 10.12669/pjms.40.1.7688

**Published:** 2024

**Authors:** Palwasha Babar, Uswa Qaiser, Ijaz ur Rehman

**Affiliations:** 1 Palwasha Babar, MDS Assistant Professor, Department of Paediatric Dentistry, Fatima Memorial Hospital College of Medicine and Dentistry, Lahore, Pakistan; 2 Uswa Qaiser, BDS Demonstrator, Department of Operative and Pediatric Dentistry, University College of Dentistry, The University of Lahore, Lahore, Pakistan; 3 Ijaz ur Rehman, FCPS Assistant Professor, Department of Oral Medicine, University College of Dentistry, The University of Lahore, Lahore, Pakistan

**Keywords:** Prescriptions, Clinical pharmacology, Dental education

## Abstract

**Objective:**

To assess the knowledge and skills of prescription writing among dental house officers from different hospitals.

**Methods:**

This cross-sectional study was conducted from July to September, 2022. A self-administered, structured questionnaire was used to collect data from 180 house officers from four teaching dental hospitals (n=45 each). The participants were asked to write a prescription for an adult and a pediatric patient. The prescription was evaluated according to WHO criteria. Analysis was done using SPSS v.20. Data was presented as frequencies and percentages.

**Results:**

Among the 180 participants, 42.9% were males and 57.1% were females. 33.9% participants reported prescription writing to be a difficult task. Only 36.7% participants reported to be trained in prescribing pediatric medications. None of the prescriptions completely fulfilled the WHO criteria. Doctor related information (name, address and contact no) was written by only 3.8% of the participants while 10% of the participants mentioned the patient related information (name, address and age). Dosage of the prescribed drugs was the most commonly drug-related missing parameter which was mentioned in 30% of the pediatric prescriptions and 21% of the adult prescriptions.

**Conclusion:**

There is a general lack of knowledge among the dental house officers regarding prescription writing as they were found to be unaware of the essential elements of a prescription. The findings call for an urgent change in the undergraduate teaching of prescription writing skills with special emphasis on pediatric drugs and dosage.

## INTRODUCTION

A prescription is a way of communication between the doctor and the pharmacist.^1^ It is a form of medical care provided by a health-care practitioner to the patient. The word prescription is of Latin origin; ‘pre’ meaning before and ‘script’ meaning writing, which means that it is an order written before or for the administration of the drug.^2^ There is no global standard for prescription writing and different countries have different laws and regulations regarding it.^3^ According to WHO guidelines, the essential elements of a good prescription include (i) date of prescription (ii) prescriber’s details: name, address and contact number (iii) patients details; name, address and age (iv) drug related information: name, strength of the drug, dosage form and total amount, frequency, length of the treatment, label instructions and warnings. However, poor adherence to these guidelines has been reported globally which results in prescription errors.

There is plenty of literature on the prescription errors by the healthcare providers. As much as 68-75% of the adverse drug reactions have been attributed to the incorrectly written prescriptions.^4^ Prescription errors may occur because of poor choice of drug, inappropriate dosage, route of administration, frequency or duration of the treatment.^5^ It may also arise due to illegible handwriting, incorrect spelling, incorrect drug knowledge and distraction or inattentiveness while writing prescription.^6^ Though rarely fatal, these errors can make it difficult for the pharmacist in dispensing correct medications resulting in ineffective treatment, prolongation of disease and distress to the patient.^2^

In a recent study conducted in Bahawalpur, Pakistan, none of the 300 prescriptions that were assessed was found in accordance with the standard guidelines or parameters.^7^ A study by Ibrahim et al., reported an astonishing evidence of 51.2% drug related problems in pediatric prescriptions.^8^ There is general consensus in the literature regarding the lack of knowledge and self-confidence in prescription competency of the medical students and newly qualified doctors_._^9^ The aim of our study was to assess the knowledge and skills of prescription writing among dental house officers from different hospitals.

## METHODS

A cross-sectional, descriptive study was conducted to assess the prescription writing skills of fresh dental graduates. Data was collected from the house officers of four private dental institutes after getting institutional permission. The participants were informed about the objectives of the study and consent was taken. The duration of the study was from July to September, 2022. The sample size was calculated to be 179, with 90% confidence level, 5.1% margin of error and by taking expected percentage of factor of interest as 22%.^10^ As the data was collected from four institutes, we divided our sample size in four strata (dental colleges) and 45 house officers were taken from each institute.

### Ethical Approval

The study was conducted in accordance with the Declaration of Helsinki. Ethical approval was taken from the Ethical Review Board of University College of Dentistry, The University of Lahore (ref no UCD/ERCA/21/11/dh).

Non-probability, convenience sampling technique was used. Dental graduates who were currently doing their house job were included irrespective of the duration of house job. Those who had completed their house job and those who did not give consent were excluded. A self-structured questionnaire was used. The first section included seven closed-ended items to assess the perception of the house officers regarding their training, confidence and difficulty in prescription writing. In the second section, the participants were asked to write a complete prescription with all necessary details of any antibiotic for an adult and a pediatric patient. The prescriptions were evaluated according to the WHO criteria.^3^ The data was analyzed using SPSS version25. Descriptive statistics were reported as percentages and mean. As the aim of the study was only to evaluate the prescription writing skills, no comparison among the dental colleges was done.

## RESULTS

A total of 180 participants (house officers) were included in the study of which 42.9% (n=77) were males and 57.1% (n=103) were females. 83.3% (n=150) participants reported to have learned prescription writing during their undergraduate program. About 33.9% (n=61) participants found prescription writing to be a difficult task while 67.8% (n=122) reported facing difficulty when prescribing medications to patients with special medical conditions. When inquired about pediatric medications, only 36.7% (n=66) participants agreed on being trained and 42.2% felt confident in prescribing pediatric medications while 88.9% (n=160) participants reported to verbally instruct the patient regarding the prescription and 81.1% (n=146) participants felt that they required additional training regarding prescription writing.

**Fig.1:**
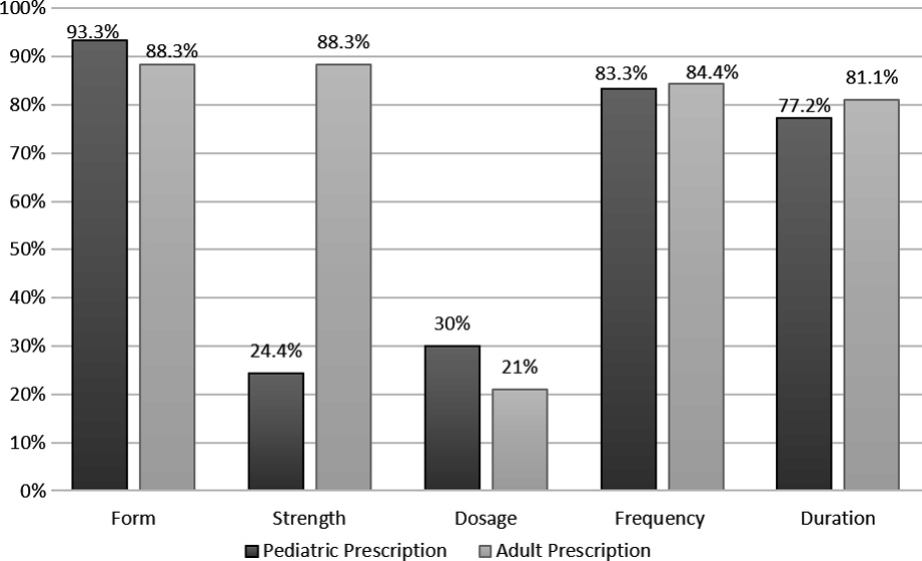
Drug related information in the prescriptions written by the participants.

None of the 180 prescriptions mentioned the WHO defined parameters completely. Doctor related information (name, address and contact no) was written by only 3.8% of the participants. 7.2% of the participants mentioned date while 10% of the participants mentioned the patient related information (name, address and age). The drug-related information as written in adult and pediatric prescriptions is summarized in Fig.1. It was found that dosage of the drugs was the most commonly drug-related missing parameter which was mentioned in 30% of the pediatric prescriptions and 21% of the adult prescriptions. The strength of the drug was mentioned in 24.4% of the pediatric prescriptions only.

**Fig.2:**
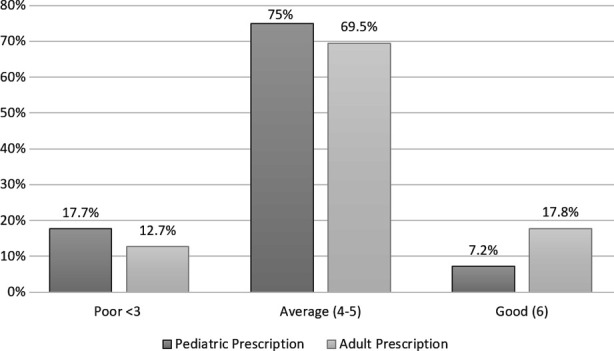
Score of the adult and pediatric prescriptions based on drug-related factors.

Since most of the participants did not mention the date and doctor/patient related factors, these parameters were not included in scoring the prescription. Each adult and pediatric prescription by the participants was scored from 1-6 on the basis of drug related information, one each for form, strength, dosage, frequency, duration and legibility. Prescriptions with a score of six was categorized as good, 4-5 as average and <3 as poor. It was found that all the drug related parameters were mentioned in only 7.2% and 7.8% of the pediatric and adult prescriptions respectively. The comparative score of adult and pediatric prescriptions is shown in Fig-2. Only 8.3% of the participants mentioned the generic name of the drugs. Regarding the legibility of the prescriptions, 97.2% were legible, 1.1% were legible with difficulty and 1.7% were not legible. 73.9% of the prescriptions were not accompanied by the prescriber’s signature.

## DISCUSSION

The rational use of medication warrants smooth and quick treatment which can only be ensured through meticulous prescription writing.^11^ The results of our study highlight the discrepancy in prescription writing skills among the dental house officers. It was found that the participants were unaware of the core elements of a standard prescription. These results are in agreement with the literature whereby poor prescribing skills of the junior doctors have been reported in numerous studies.^12^,^13^ A study conducted in Karachi, Pakistan highlighted the deficiency in prescription writing skills and poor adherence to WHO guidelines among the dental house officers.^10^ A recent survey showed that as much as 50.5% of the dental students were unaware of WHO guidelines of prescription writing.^14^

Participants reported a low confidence in writing pediatric prescriptions and only 36.7% said to be trained about pediatric medications. This was also reflected in the written pediatric prescriptions as only 7.2% of the prescriptions had drug related parameters completely mentioned. It was observed that the strength and the dosage of the prescribed drugs were the most common missed elements. This may be due to the lack of knowledge regarding the pediatric medications, their available strengths and dosages. A study regarding drug prescription among the dental residents showed that 44.28% of the residents lacked knowledge about drug posology.^15^ Another study by Wroblewski et al. reported that neither a weight-based dosing standard nor the patient’s weight was mentioned on as many as 98% prescriptions.^16^

A contrasting trend of writing trade name for drugs instead of generic name was seen in our study which is against the recommendation of WHO.^6^ Only 25.5% of the participants wrote generic names. In a study conducted in India, only 8.3% participants wrote generic name for drugs while writing a prescription.^17^ Similar trend was noticed in another study conducted in Karachi where only 4% of the house officers prescribed generic drugs names. A survey conducted in Nepal revealed that the doctors apprehend that prescribing generic medicines is less effective and causes more side effects than the brand name medicines.^18^ This misconception needs to be addressed in order to ensure optimal drug prescription.

Majority of the participants expressed that they require additional training regarding prescription writing. The results highlight the inadequate training in the domain of clinical pharmacology during the undergraduate program. Medical and dental students learn prescription writing in their pre-clerkship years. A comprehensive study across Europe also pointed that the undergraduate curriculum had not adequately prepared the young doctors for safe prescribing. There is a dire need to revise the undergraduate curriculum in a way which helps to improve prescribing competency among the students. Different approaches have been proposed in this regard. A study proposed that junior doctors should be co-working with pharmacists and consultants in order to improve prescribing practices.^19^ Case-based learning and simulated clinical encounters using structured videos.^20^,^21^ These approaches should be incorporated in the curriculum so that students master this essential skill.

### Limitations

The results are based on the number of parameters mentioned in a prescription and not on the accuracy of the prescription. A score was given if the parameters were mentioned even if it was inaccurate.

## CONCLUSION

There is a general lack of knowledge among the house officers regarding prescription writing as the house officers were found to be unaware of the essential elements of a prescription. The findings call for an urgent change in the undergraduate teaching of prescription writing skills with emphasis on pediatric drugs and dosage.
